# Case report: *IKZF1*-related early-onset CID is expected to be missed in TREC-based SCID screening but can be identified by determination of KREC levels

**DOI:** 10.3389/fimmu.2023.1257581

**Published:** 2023-09-12

**Authors:** Christofer Äng, Rolf H. Zetterström, Kim Ramme, Emma Axelsen, Per Marits, Mikael Sundin

**Affiliations:** ^1^ Sachs Children’s Hospital, Södersjukhuset, Stockholm, Sweden; ^2^ Division of Pediatrics, Department of Clinical Science, Intervention and Technology, Karolinska Institutet, Stockholm, Sweden; ^3^ Center for Inherited Metabolic Diseases, Medical Diagnostics Center, Karolinska University Hospital, Stockholm, Sweden; ^4^ Division of Inborn Errors of Endocrinology and Metabolism, Department of Molecular Medicine and Surgery, Karolinska Institutet, Stockholm, Sweden; ^5^ Department of Pediatric Hematology and Oncology, Children’s Hospital, Uppsala University Hospital, Uppsala, Sweden; ^6^ Department of Women's and Children's Health, Uppsala University, Uppsala, Sweden; ^7^ Section of Pediatric Hematology, Immunology and HCT, Astrid Lindgren Children’s Hospital, Karolinska University Hospital, Stockholm, Sweden; ^8^ Department of Clinical Immunology, Medical Diagnostics Center, Karolinska University Hospital, Stockholm, Sweden

**Keywords:** SCID, IKZF1, Ikaros, newborn screening, CID, HCT (hematopoietic cell transplant)

## Abstract

This report illustrates a case that would have been missed in the most common screening algorithms used worldwide in newborn screening (NBS) for severe combined immunodeficiency (SCID). Our patient presented with a clinical picture that suggested a severe inborn error of immunity (IEI). The 6-month-old baby had normal T-cell receptor excision circle (TREC) levels but no measurable level of kappa-deleting recombination excision circles (KRECs) in the NBS sample. A *de novo IKZF1-*mutation (c.476A>G, p.Asn159Ser) was found. The clinical picture, immunologic workup, and genetic result were consistent with *IKZF1*-related combined immunodeficiency (CID). Our patient had symptomatic treatment and underwent allogeneic hematopoietic cell transplantation (HCT). *IKZF1*-related CID is a rare, serious, and early-onset disease; this case provides further insights into the phenotype, including KREC status.

## Introduction

IEI are a diverse group of monogenic diseases characterized by a malfunctioning immune system. In recent years, screening for the most fatal form, SCID, has been implemented in various settings. Before the start of NBS for SCID, these patients commonly present with infections in their first 6 months of life, and because of their pronounced immunodeficiency, were unable to manage these infections. If left untreated, most succumbed within their first 2 years of life (1, 2). An early diagnosis has been associated with favorable outcome (3). Hence, in recent years NBS by measuring TREC levels using Guthrie cards has been implemented in many countries, including Sweden (4). TREC-based SCID NBS is expected to and has been demonstrated to identify other potential life-threatening T lymphopenic conditions, e.g., CID not fulfilling SCID criteria and thymic defects (5). The addition of KREC analysis in SCID NBS has been explored in research aiming at identifying serious CID and B cell immunodeficiency (6).

One IEI-associated gene is *IKZF1*. The IKZF1 protein is a member of the family of zinc-finger proteins (IKZF1-IKZF5) that bind DNA through four N-terminal zinc-finger domains and dimerize through two C-terminal zinc-fingers (7). It targets DNA sequences at pericentromeric heterochromatin regions of certain genes and regulates the nucleosome remodeling and histone deacetylase complex, and through that process either activates or represses transcription (8). IKZF1 is well-studied in its role as a regulator of hematopoiesis, with it being crucial for lymphocyte development, differentiation, and tumor suppression. Mutations in the gene have previously been reported to cause CID (9, 10).

We present a child with a *de novo* point mutation in *IKZF1*, resulting in a clinically severe form of early-onset CID. The child was born just before the Swedish introduction of SCID NBS but presented when national TREC-based NBS was up and running. Testing of the newborn sample at presentation was normal by TREC but pathologic by KREC levels.

## Case description

**Figure 1:**
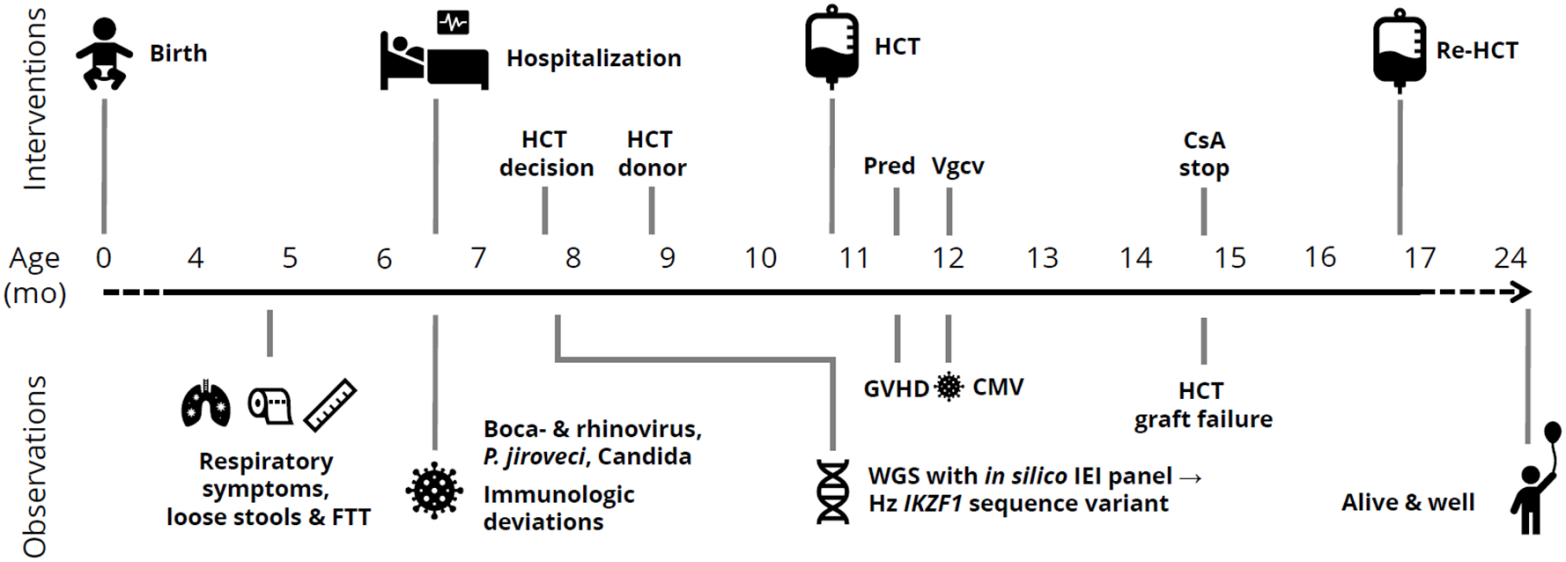
Timeline of clinical observations and interventions in a child with *IKZF1*-related CID. mo, months; FTT, failure-to-thrive; HCT, allogeneic hematopoietic cell transplantation; Pred, prednisolone; Vgcv, valganciclovir; GVHD, graft-versus-host disease; CMV, cytomegalovirus; IEI, inborn errors of immunity; Hz, heterozygous; CsA, ciclosporine A.

At 6-month-old baby girl, born to non-consanguineous parents of Swedish descent (Caucasians), was admitted to a Swedish hospital due to severe pneumonia. Previous medical records showed failure-to-thrive and thrombocytosis. She received respiratory support, corticosteroids, and antibiotics at the intensive care unit. Rhino- and bocavirus were detected in nasal swabs, and radiology suggested a bacterial infection. Due to halting recovery after a week, extended testing was performed, revealing unmeasurable immunoglobulin levels and a nasopharyngeal swab PCR positive for *Pneumocystis jirovecii* (PCJ). Radiology and further microbiologic testing confirmed PCJ pneumonia. This raised a suspicion of SCID, and antimicrobial therapy was adjusted and intravenous immunoglobulin substitution initiated. The patient was transferred to a tertiary pediatric hospital for intensive care and immunologic workup and treatment. *Candida parapsiolosis* was found in the feces. The necessity of allogenic HCT was evident; thus, it was carried out. The timeline is depicted in **Figure 1**.

## Diagnostic assessment

Upon clinical diagnosis, we retrieved the patient’s NBS sample and ran an SCID NBS assay analyzing both TRECs and KRECs: the TREC copy number was 110 copies/well [recall threshold ≤5, population mean 79, IQR 55, 110 (4)] and KRECs were not measurable. Lymphocyte phenotyping in peripheral blood showed prevalent T lymphocytes of which the majority were CD4^+^ T lymphocytes, with a smaller fraction of CD8^+^ T lymphocytes. Both CD4^+^ and CD8^+^ T lymphocytes had a uniform, atypical surface phenotype expressing intermediate levels of the naïve cell marker CD45RA but high levels of the recent thymic emigrant marker CD31 (**Table 1**). Analysis of the T lymphocyte receptor (TCR) Vβ repertoire by flow cytometry revealed an essentially normal distribution in both CD4^+^ and CD8^+^ T lymphocytes, but an elevated level of CD4+ Vβ2^+^ T lymphocytes (19%, upper normal limit 13%). B lymphocytes were severely reduced in numbers (0.02 × 10^9^/L) and were all found to be naïve (93% with no plasmablasts and switched memory cells, **Table 1**). No specific antibodies to previous vaccinations were detected. Lymphocyte proliferation showed adequate response to mitogens, except for pokeweed mitogen (PWM) in B lymphocytes and concavalin A (ConA) in CD8^+^ T lymphocytes. Conversely, a lymphocyte response was not evoked to either tetanus or pneumococcus despite previous vaccinations, or candida despite positive cultures (**Table 1**). Assessment of the myeloid compartment (e.g., erythro-, mono-, and neutrophil granulocytes) was normal, besides absent eosinophil granulocytes and thrombocytosis (**Table 1**). Whole genome sequencing with an *in silico* filter for IEI (11) showed a heterozygous sequence variant in *IKZF1* (c.476A>G, p.Asn159Ser). Confirmatory Sanger sequencing of the patient and parents showed the mutation to be *de novo*.

**Table 1:** Hematologic and immunologic findings in a child with *IKZF1*-related CID.

	At diagnosis (6 mo old)	Post-HCT 1^st^(13 mo old)	Post-HCT 2^nd^ (16 mo old)	Follow-up (22 mo old)
Full blood count				
**Hemoglobin (g/L)**	128	115	94 ↓	116
**Leukocytes (10^9^/L)**	14.8	3.6 ↓	1.0 ↓	9.1
Neutrophils	6.4 ↑	3.3	0.9	4.2
Eosinophils	0 ↓	0.1	<0.1	0.1
Basophils	0.03	<0.1	<0.1	<0.1
Monocytes	0.5	<0.1 ↓	0.1 ↓	0.7
Lymphocytes	7.8 ↑	0.2 ↓	0.9 ↓	4.6
**Thrombocytes (10^9^/L)**	720 ↑	246	271	327
T lymphocytes				
CD3^+^ (10^9^/L)	4.61	0.09 ↓	1.76	3.14
CD4^+^ (10^9^/L)	3.78	0.04 ↓	0.37 ↓	0.51 ↓
CD8^+^ (10^9^/L)	0.32 ↓	0.04 ↓	1.34	2.58
CD4^+^ CD45RA^+^ CCR7^+^ (naïve, %)	>95 ↑ #	-	-	35
CD4^+^ CD45RA^+^ CD31^+^ (RTE, %)	>95 ↑ #	–	–	–
CD4^+^ CD45RA^-^ CCR7^-^ (EM, %)	↓ #	-	-	23
CD4^+^ CD45RA^-^ CCR7^+^ (CM, %)	↓ #	–	–	35
CD4^+^ CD45RA^+^ CCR7^-^ (EMRA, %)	↓ #	-	-	7
CD8^+^ CD45RA^-^ CCR7^-^ (EM, %)	↓ #	–	–	25
CD8^+^ CD45RA^-^ CCR7^+^ (CM, %)	↓ #	-	-	1
CD8^+^ CD45RA^+^ CCR7^-^ (EMRA, %)	↓ #	–	–	72
Lymphocyte proliferation (counts/µL)				
CD4+ PWM	2145 ↑	–	–	–
CD8+ PWM	633 ↓	-	-	-
CD19^+^ PWM	1 ↓	–	–	–
CD4^+^ ConA	822	-	-	-
CD8^+^ ConA	144 ↓	–	–	–
CD4^+^ Pneumococcus	0 ↓	-	-	-
CD8^+^ Pneumococcus	0 ↓	–	–	–
CD4^+^ Tetanus toxin	0 ↓	-	-	-
CD8^+^ Tetanus toxin	0 ↓	–	–	–
CD4^+^ Candida	0 ↓	-	-	-
CD8^+^ Candida	0 ↓	–	–	–
B lymphocytes				
CD19^+^ (10^9^/L)	0.02 ↓	0.01 ↓	0.01 ↓	0.13 ↓
CD19^+^ IgD^+^ CD27^-^ (naïve, %)	93 ↑	-	-	-
CD19^+^ IgD^-^ CD27^+^ (SM, %)	<0.5 ↓	–	–	–
NK cells				
CD16^+^ CD56^+^ (10^9^/L)	0.07 ↓	0.14 ↓	0.12	0.12
Immunoglobulin levels				
IgG (g/L)	<0.1 ↓	3.8	5.9	7.4
IgA (g/L)	<0.08 ↓	<0.08 ↓	0.24	0.27
IgM (g/L)	<0.1 ↓	<0.1 ↓	0.13 ↓	0.35
IgE (IU/L)	<2.00 ↓	–	–	–
Pneumococcus IgG (mg/L)	<0.01 ↓	–	–	–
Tetanus IgG (IU/mL)	<0.1 ↓	-	-	-
Chimerism (% recipient)				
CD3^+^	-	3.8	0.01	0
CD19^+^	–	1.6	0.02	0
CD33^+^	-	79.9	0.02	0

HCT, hematopoietic cell transplantation; mo, months; RTE, recent thymic emigrants; EM, effector memory; CM, central memory; EMRA, exhausted effector memory; PWM, pokeweed mitogen; ConA, concanavalin A; SM, switched memory; ↓, below normal values; ↑, above normal; #, inconclusive result where all T lymphocytes express intermediate levels of CD45RA and CD31^+^.

## Therapeutic intervention

At 10 months of age, our patient underwent allogeneic HCT with an unrelated 10/10 HLA-matched donor, the timeline is depicted in **Figure 1**. The PCJ pneumonia and respiratory viruses were cleared before admission. HCT conditioning consisted of fludarabine, treosulfan, and anti-thymocyte globulin. The HCT was performed with neutrophil and thrombocyte engraftment at days +36 and +24, respectively, without complications. Ciclosporin A and methotrexate were used as graft-versus-host disease (GVHD) prophylaxis. Six weeks after the procedure, cutaneous and gastrointestinal GVHD grade 1 occurred and was treated with prednisolone. Thereafter, CMV viremia occurred, with good response to pre-emptive therapy with valganciclovir. Because of mixed chimerism with low counts of T and B lymphocytes, an evaluating bone marrow biopsy was performed approximately +4 months post-HCT. It revealed near total CD34^+^ autologous reconstitution, i.e., graft failure/rejection. Hence, re-HCT was performed with repeated conditioning (i.e., same as in first HCT with the addition of thiotepa) and the same donor and GVHD prophylaxis. Neutrophil and thrombocyte engraftment were seen at days +19 and +20, respectively. The re-HCT was without significant complications.

## Follow-up and outcomes

Six months after re-HCT, the patient displayed full donor chimerism. An additional 12 months later, our patient experienced several uncomplicated upper airway infections, with spontaneous recovery. Twenty-seven months after re-HCT, the patient is alive and well, without any continuous treatment. She has a full numeric immune recovery (lymphocytes and immunoglobulins), responded adequately to vaccinations, is developing as expected, and is now in preschool.

## Discussion

Our patient presented with evident IEI caused by a dominant negative mechanism providing loss of function in *IKZF1*, resulting in faltering lymphocyte development. This mechanism of action has previously been reported in presenting phenotypes similar to our patient’s (12). The result is early-onset *IKZF1*-related IEI with a severe clinical phenotype (infections), characterized by somewhat functioning T lymphopoiesis (TREC generation and normal T lymphocyte counts), haltered T lymphocyte responses and memory generation, and a pronounced B lymphocyte defect. Although our patient’s phenotype was presumably fatal if left untreated, and hence the CID could be regarded as severe, our patient did not meet the SCID criteria according to the Primary Immune Deficiency Treatment Consortium (PIDTC) 2022 Definitions for SCID (13). The criteria are based on absolute T lymphocyte counts, not taking immune function and clinical phenotype into account. However, naming the disease SCID or pronounced early-onset CID might not impact the clinical care.

*IKZF1*-related CID has been described among at least seven patients previously reported by Boutboul et al. (10). Their phenotypes showed many similarities to our patient’s, i.e., CID with lack of T lymphocyte activation and memory generation (e.g., abnormal T lymphocyte population with intermediate CD45RA expression and CD31^+^) and low B lymphocyte levels, the evident major features of *IKZF1*-related CID. Boutboul et al. assessed TREC levels in three of their patients and they were normal to high (10), as in our patient, who had normal TREC levels. Our patient had a pronounced B lymphocyte development arrest, as seen in two of the patients in the Boutboul et al. cohort (10). The B lymphocyte arrest was reflected by the absence of KRECs, which has not been previously described in *IKZF1*-related CID. Additionally, we showed that our patient’s B lymphocytes did not respond to PWM stimulation. Our patient contrasts to the previously reported ones with increased CD4^+^ Vβ2^+^ T lymphocytes, severely reduced CD8^+^ T lymphocytes, reduced CD8^+^ T lymphocytes proliferative response to mitogen (ConA), and no clear myeloid defects besides the unmeasurable eosinophils. However, regarding the latter, a slight myeloid defect might have been disguised by an inflammatory response to severe infection (i.e., PCJ pneumonia and respiratory tract virosis).

*IKZF1*-associated disease has previously been identified through TREC-based SCID NBS (9). However, our patient presented with normal levels of TREC and unmeasurable levels of KREC. The level of T lymphocytes was evidently enough to produce TRECs, and yet, presumably, this *IKZF1*-mutation provided a CD4^+^ T lymphocyte dysfunction that led to an absence of both CD8^+^ T and B lymphocytes. Additionally, there also seems to be an intrinsic B lymphocyte defect, as previously reported (14). In total, the combination of T and B lymphocyte dysfunction resulted in an absence of KRECs. The latter is not included in the Swedish national NBS program (4). It was, however, measured in this patient as a residual part of a research program (pilot) for SCID NBS and was hence available (6). If KREC analysis had been included in the NBS program, this patient would presumably have been detected at a much earlier stage. Early diagnosis is a strong contributing factor to a positive outcome for patients with severe early-onset IEI, wherefore including KREC determination in the NBS program would presumably improve the outcome for future patients with similar conditions. However, including KREC analysis in SCID NBS comes with a higher recall ratio and poor positive predictive values (6). This leads to a lot of stress for families of children with abnormal NBS samples that, after diagnostic workup, turn out to be normal or were reversibly abnormal due to maternal medication. Second tier testing with NGS, as is practiced in some countries in the case of low TRECs, might be an option to tackle these downsides (15, 16). In the case of *IKZF1*-related CID, lymphocyte phenotyping (i.e., abnormal T lymphocytes with intermediate CD45RA expression and CD31^+^) as second tier or primary diagnostics could be a valuable tool in identifying these patients.

Our patient was successfully engrafted after re-HCT and displayed a full numeric immune recovery, with good response to vaccinations and lives a normal life. Hence, the profound CID seems to have been cured by the intervention. In mouse models of *IKZF1*-related CID, GVHD severity and HCT-related mortality were considerably high, so Kellner et al. compiled the first four known patients undergoing HCT due to *IKZF1*-related CID. These patients had a similar HCT outcome compared to our patient (i.e., upper normal to slightly delayed engraftment, treatable CMV viremia; 2/4, curable GVHD; 1/4, and full immunohematopoietic recovery in all surviving; 3/4) (17). Our patient rejected her first HCT graft after intermediate intensive conditioning. A rejection (1/4) without conditioning was also seen in the Kellner et al. cohort. These two rejecting patients may indicate that more intensive conditioning is needed in *IKZF1*-related CID, as used in our re-HCT and other reported engrafted patients (17).

As discussed, there are advantages and disadvantages to KREC determination in SCID NBS; however, this case illustrates the positive impact an inclusion of KRECs would have for patients with life-threatening early-onset CID.

## Patient perspective

Today, our daughter looks and acts just as any three-year-old child on the outside. But it has not always been like this. Her life began brutal, with incredibly tough challenges and suffering. First and foremost, for her, but also for us as first-time parents, including everyone in our circle of acquaintances. There are no words that can describe how it feels as a parent when you think you are about to lose your child. If she could have been diagnosed earlier through NBS, we could have prevented severe and life-threatening infections, months of hospitalization, and shortened the already long time to recovery and eventually to the declaration of health. With that being said, our wish is that no other child and no family ever have to go through what we went through. Therefore, it is our sincere hope that all abnormalities that are linked to treatable serious diseases should be included in the NBS.

## Data availability statement

The original contributions presented in the study are publicly available. This data can be found here: https://www.ncbi.nlm.nih.gov/clinvar/; RCV003326142.1.

## Ethics statement

The studies involving humans were approved by Swedish Ethical Review Authority. The studies were conducted in accordance with the local legislation and institutional requirements. Written informed consent for participation in this study was provided by the participants’ legal guardians/next of kin. Written informed consent was obtained from the individual(s), and minor(s)’ legal guardian/next of kin, for the publication of any potentially identifiable images or data included in this article.

## Author contributions

CÄ: Investigation and Writing – original draft. RZ: Resources and Writing – review & editing. KR: Writing – review & editing. EA: Writing – review & editing. PM: Resources and Writing – review & editing. MS: Conceptualization, Funding acquisition, Supervision and Writing – review & editing.
